# Supplementary material for: Analysis of the recombination landscape of hexaploid bread wheat reveals genes controlling recombination and gene conversion frequency
Source: Genome Biol. 2019 Apr 15;20:69. doi: 10.1186/s13059-019-1675-6 (PMC6463664; doi:10.1186/s13059-019-1675-6)
Supplement: Supplementary file 1 — Includes supplemental Information in PDF format including Supplemental Notes S1-S9, Supplemental Figures S1-S9 and Supplemental Tables S1-S7. (PDF 6178 kb) [file 13059_2019_1675_MOESM1_ESM.pdf]

Supplemental Information

Supplemental Notes

Note S1-note S7

Supplemental Figures

Figure S1- Figure S9

Supplemental Tables

Table S1-Table S7

Supplemental Notes

Note S1: For the Holliday junction ATP-dependent DNA helicase *RuvB-like* that was associated with CO frequency, we identified two lines with likely knockouts via introduced stop codons and a further six with missense mutations (Supplemental Table S6). We defined CO frequency for each of the eight mutant lines using SNPs that were defined from the TILLING population exome capture data (CO-Phenotype, Methods). To enable comparison, we identified ten control lines from the TILLING population with no mutations in our genes of interest and calculated their CO frequencies (CO-Phenotype). After comparison, the CO frequencies of the knockout *RuvB* lines and control group largely overlapped with average frequencies of 57.4 and 57.6 respectively (Two tailed t test, P=0.9562, t=0.0558, df=16) (Figure 4a, Methods).

Note S2: For *RecQ-7* that was associated with GC frequency, we identified four lines with likely knockouts via introduced stop codons (Supplemental Table S6) and defined GC frequency for each of the four mutant lines (GC-Phenotype, Methods). We used the same control lines for comparison as used for the *RuvB* analysis but this time calculated their GC frequencies for the lines.

Note S3: The average number of SNPs that were available to perform our GC phenotyping analysis across the Cadenza TILLING lines under analysis was 5,207 with a range from 1,700-9,018. Following on from this 462.5 was the average number of CO/GCs that were identified across the TILLING lines with 405.5 likely GCs. Previously, using the array SNPs, with on average 4335 SNPs available for analysis per population, we defined an average of only 104 GCs per RIL across the 13 populations (Supplemental Table S2). This increase in GC detection for the TILLING lines is thought to be due to a combination of the increased number of SNPs for analysis alongside the larger population size of the TILLING population (1,200 lines compared to the average size of 158 RILs for the 13 previously analyzed populations). This is supported by our identification of 335.5 GCs per RIL in the Paragon x Chinese Spring population where we have a larger number of 8,369 SNPs available and a larger population size than many of the the other RIL populations at 269.

Note S4: From Figure 4d, we were able to identify the three homoeologous wheat homologs of the *Arabidopsis* recombination candidate genes *RecQ4A* and *RecQ4B*. We then used the Cadenza TILLING population, to ascertain if knockouts of these homoeologous genes showed CO frequency phenotypes. We were able to identify 18 knockouts across the three homoeologs (Supplemental Table S6). Knockouts of our gene candidates resulted in a decrease in the average GC frequency per line from 498.5 in the control group to 413.7, 460.7 and 446.9 for sub-genome A, B and D respectively (GC-Phenotype), although only the decrease from knockouts of sub-genome A was statistically significant (Two tailed t test, sub-genome A; P=0.0163, t=2.6833, df=16, sub-genome B; P=0.3710, t=0.9243, df=14, sub-genome D; P=0.2533, t=1.2466, df=7). Looking at CO frequency, knockouts of our gene candidates resulted in a decrease in the average CO frequency per line from 59 in the control group to 53.5, 53.5 and 54.8 for sub-genome A, B and D respectively (CO-Phenotype), although none of the decreases were statistically significant (Two tailed t test, sub-genome A; P=0.1240, t=1.6236, df=16, sub-genome B; P=0.2426, t=1.2200, df=14, sub-genome D; P=0.3610, t=0.9496, df=12).

Note S5: From Figure 4d, we were able to identify the homoeologous B and D sub-genome copies of our candidate gene *RecQ-7* on chromosome 2A. There was an additional low confidence gene that was also closely related to our homoeologous trio and observed on chromosome 2D. We then used the Cadenza TILLING population, to ascertain if knockouts of these homoeologous genes showed similar phenotypes that were observed with the knockout on chromosome 2A. We were able to identify three knockouts of the homoeolog on chromosome 2B, one knockout of the homoeolog on chromosome 2D and an additional knockout of the closely associated low confidence gene on chromosome 2D (Supplemental Table S6). Knockouts of our gene candidates resulted in a decrease in the average GC frequency per line from 498.5 in the control group to 414.4 and 449.4 for sub-genomes B and D respectively (GC-Phenotype). Although a decrease was observed (Supplemental Figure S9), it was not statistically significant, potentially due to the low number of knockouts under analysis (Two tailed t test, sub-genome B; P=0.1504, t=1.5457, df=11, sub-genome D; P=0.095, t=1.8656, df=9).

Note S6: For GC frequency (as per GC-Phenotype, Methods) we identified multiple robust QTL that were seen in populations other than the Paragon x Chinese spring population. Firstly, we identified ATP-dependent DNA helicase *PIF2* from the Paragon x CIMMYT 47 analysis, for this gene we were unable to identify any Cadenza TILLING lines showing knockouts. We were able to identify two TILLING lines showing missense mutations with sift scores <0.05 in this gene, however, these mutants showed an average GC frequency of 486.5 which, although lower than the control group (498.5), was not significantly different (Welch two tailed t test, p=0.8973, t=0.1628, df=1) (Supplemental Table S7).

Secondly, we identified a gene encoding the protein HIRA from the Paragon x Watkins 94 analysis. For this gene we were able to identify six TILLING lines with potential knockouts i.e. stop codons gained (Supplemental Table S7). These mutants showed an average GC frequency of 439.4 which, although lower than the control group (498.5) by almost 60 GCs, was not significantly different (Welch two tailed t test, p=0.1632, t=1.4945, df=11).

Finally, we identified the WPP domain-interacting protein 1 from the Paragon x Baj analysis. For this gene we were unable to identify any Cadenza TILLING lines showing knockouts. However, we were able to identify seven TILLING lines with missense mutations with sift scores <0.05 (Supplemental Table S7). These mutants showed an average GC frequency of 454.5 which, although again lower than the control group (498.5), was not significantly different (Welch two tailed t test, p=0.2069, t=1.3235, df=14). Interestingly, when we went on to define CO frequency for these mutants to determine if our GC-Phenotype could translate to CO frequency, we observed a significant decrease in CO frequency in the mutant group compared to the control group (Welch two tailed t test, P=0.0277, t=2.4562, df=14) with average CO frequencies of 50.7 and 59 for mutant and control respectively. Therefore, this gene could be an important candidate for further analysis.

Note S7: The candidate gene for our third QTL encoded the protein HIRA on chromosome 4B that is needed for chromatin reassembly during DSB repair (Li, X. and Tyler, J.). HIRA mediates DNA synthesis-independent nucleosome assembly and in mammalian cells has been shown to take over for nucleosome assembly on newly replicated DNA in the absence of CAF-1 (Brachet et al., 2015). While, CAF-1 interacts in a DNA damage-dependent manner with the ATP-dependent RNA helicase RecQ (Bernstein et al., 2014; Hoek et al., 2011). This QTL therefore may highlight, within the Paragon x Watkins 94 population, the

use of an alternative but complementary pathway to the previously defined QTLs for recombination.

Our final QTL identified the WPP domain-interacting protein 1 on chromosome 5A as a candidate gene alongside the Regulator of chromosome condensation (RCC1) gene. WPP domain-interacting protein 1 mediates and enhances nuclear envelope docking of RANGAP proteins; the cycling of Ran between its GTP- and GDP-bound forms is catalysed by (RCC1) and RANGAP resulting in an intracellular concentration gradient of RanGTP that acts as a GPS for cells with regard to meiotic spindle formation that can affect chromosome segregation (Zhao, et al., 2008; Kalab and Heald, 2008; Cesario and McKim, 2011). Furthermore, Cadenza TILLING lines showing missense mutations for this gene showed a drop in GC frequency that translated to a significant drop in CO frequency. It is evident from all our QTL analyses, that the maximum peak in our defined QTL interval is consistently within 1Mbp of a functionally relevant gene that has previously been linked to either homologous recombination or DSB repair and these genes represent potential targets for wheat breeding programs in the drive to increase recombination rate.

Supplemental Figures

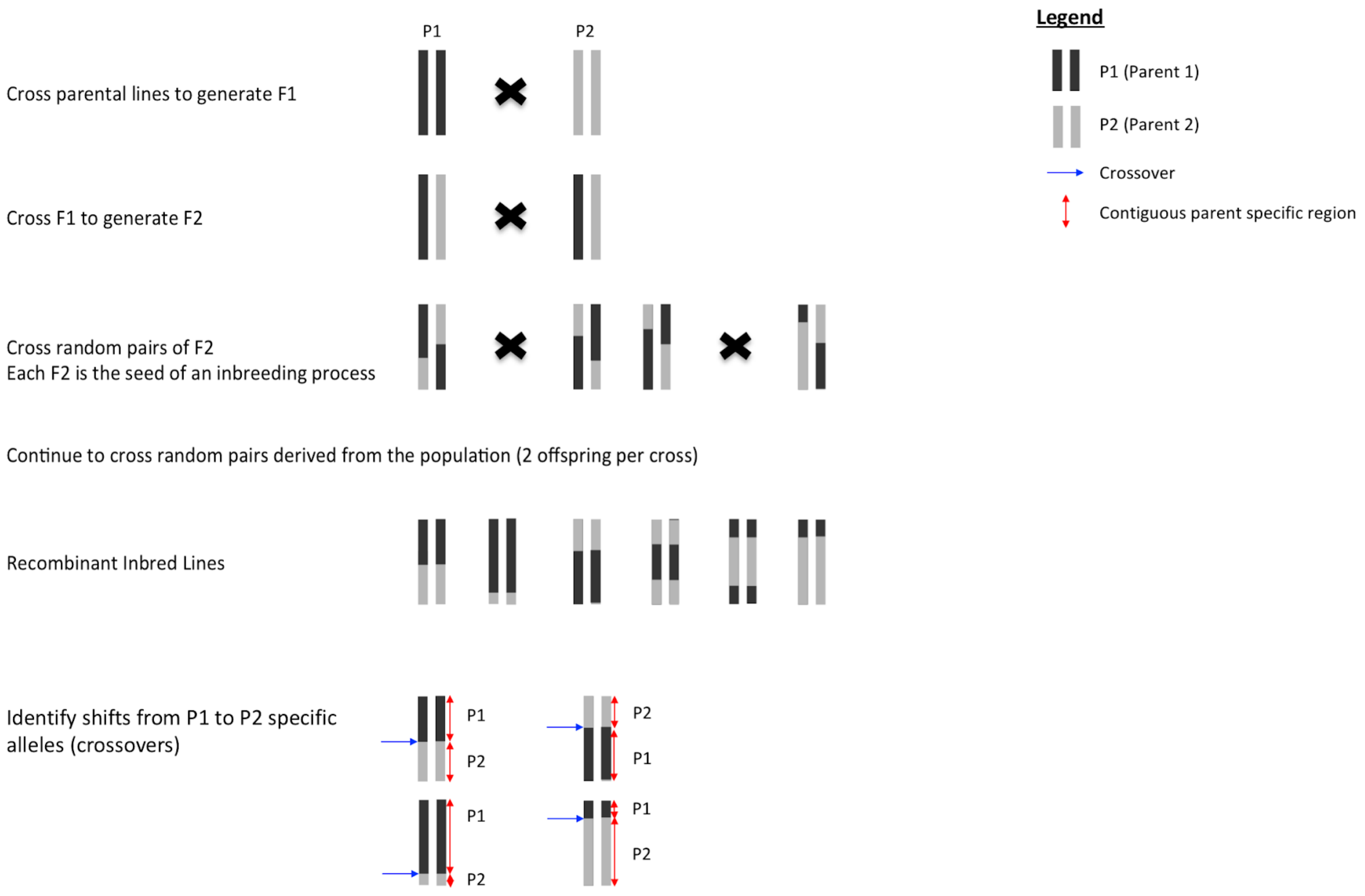

Figure S1. Construction of a RIL line and CO identification. Example of the construction of a Recombinant inbred line (RIL) where a set of diploid chromosomes represents an individual and each parental genotype is represented by either black or grey. Each individual resultant RIL is a mosaic of the parental lines with widespread homozygosity between chromosome pairs. By comparing the genotype of each RIL to that of its parental founders, it is possible to see shifts in genotype from alleles specific to Parent 1 to alleles specific to Parent 2. These shifts can be used as an estimate of recombination points or chromosomal COs across the genome to predict where they occurred during population generation.

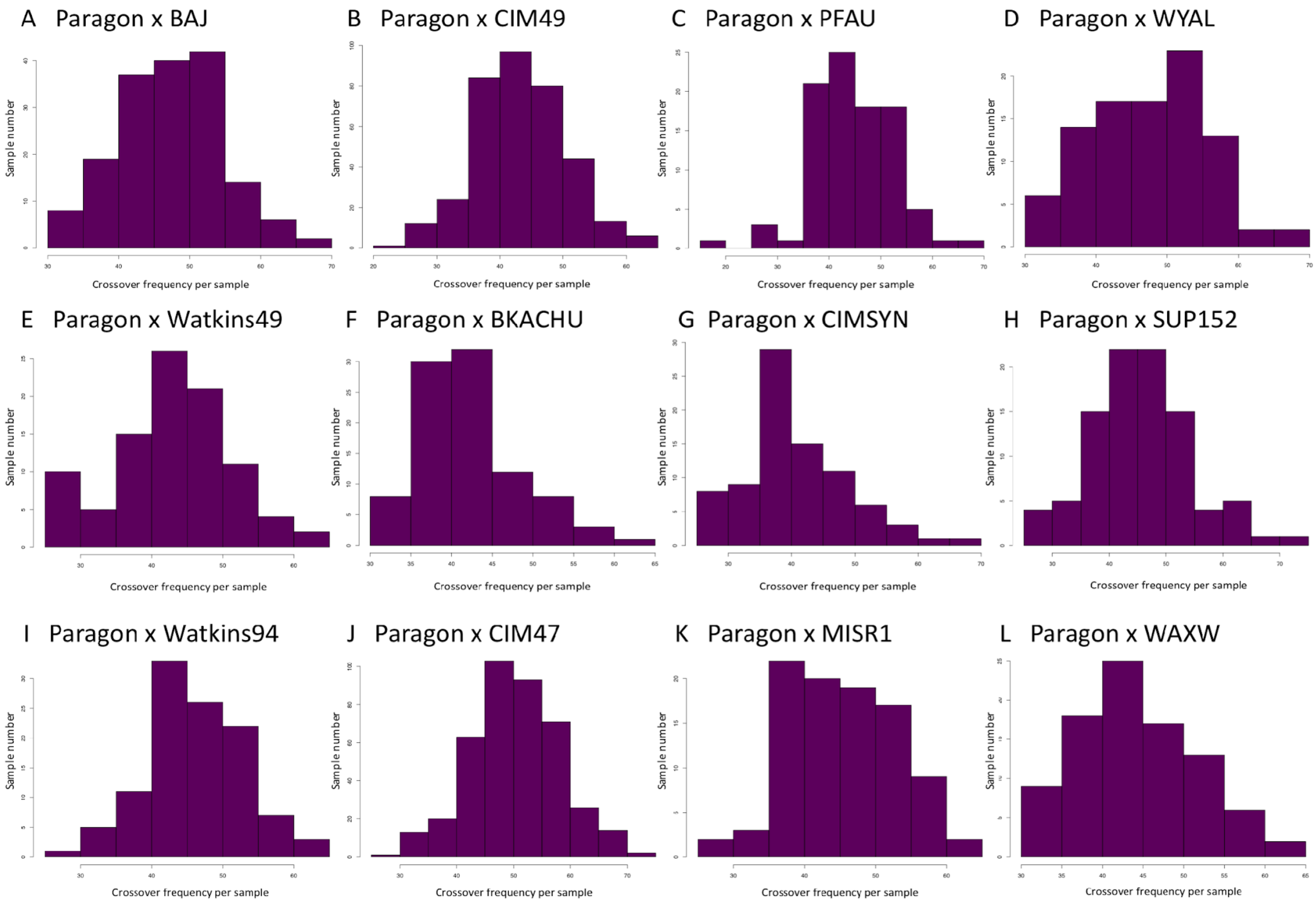

Figure S2. Frequency of COs per RIL. For each of the populations under analysis figures A-L represent the number of COs recorded for each RIL (CO frequency per sample) as a frequency histogram.

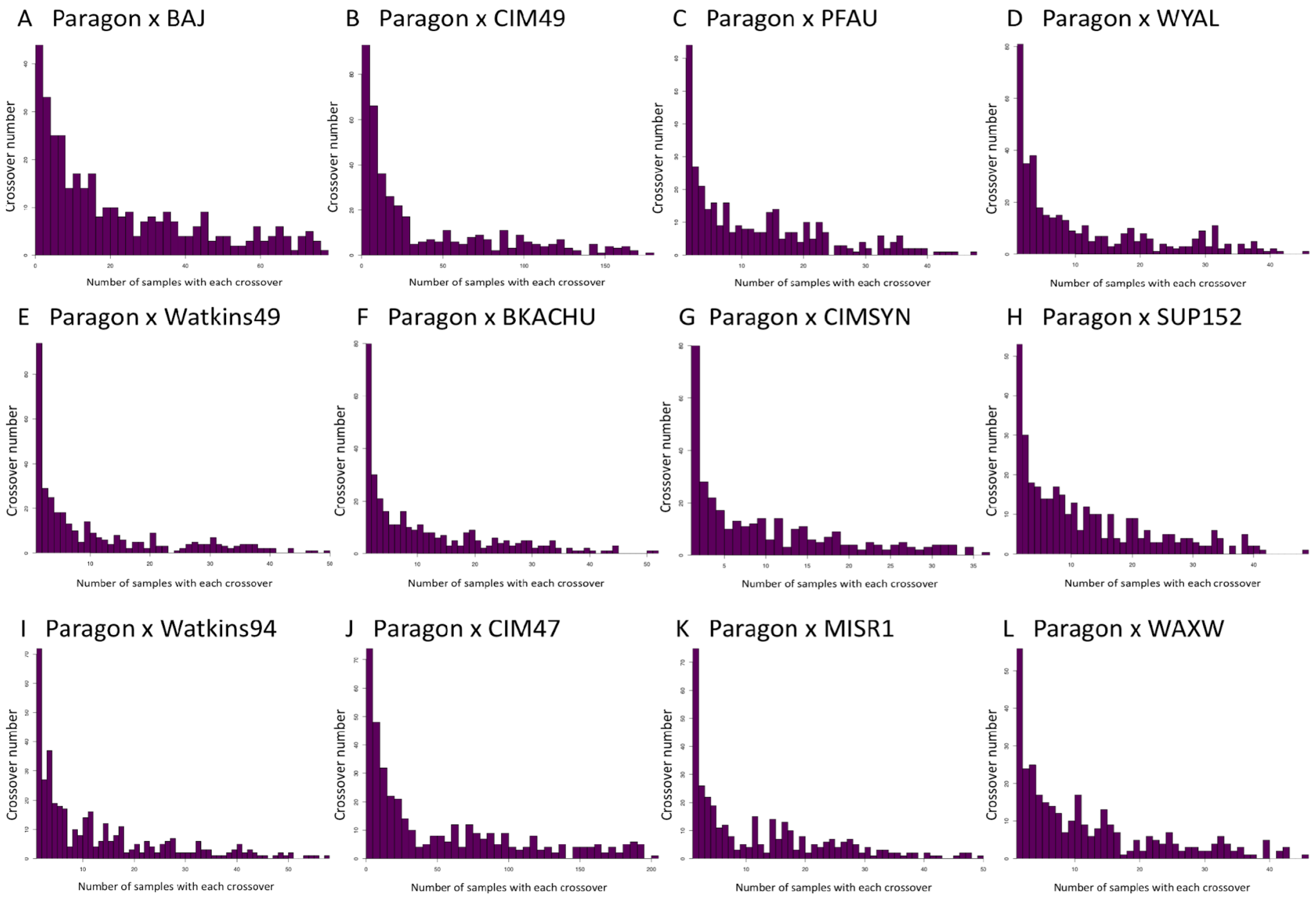

Figure S3. Number of shared COs per population. For each of the populations under analysis figures A-L represent the number of RILs sharing each recorded CO (Number of samples with each CO) as a frequency histogram.

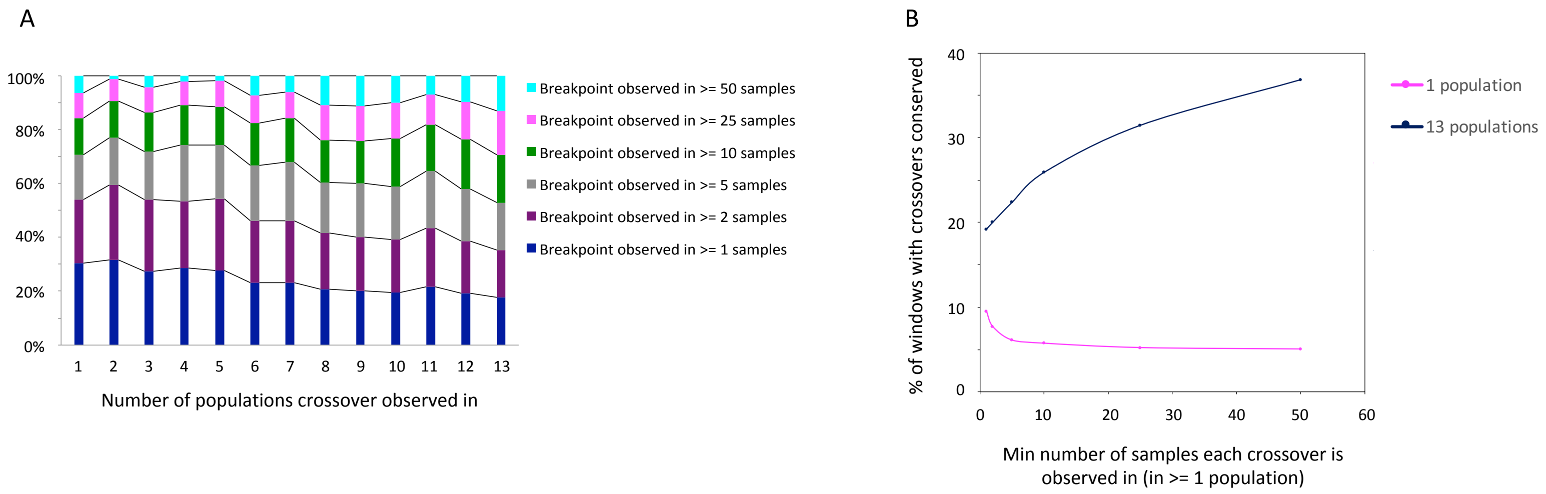

Figure S4. CO conservation between populations. (A) Stacked bar chart summing all observed COs in each category (number of populations from 1-13). Bar charts are sub divided into coloured blocks representing how many RILs shared the CO within a population (see legend). (B) Scatter Plot displaying the minimum number of RILs each CO has to have been observed in (x axis) versus the percentage of COs that are conserved (y axis) across the relevant number of populations (1 and 13 respectively).

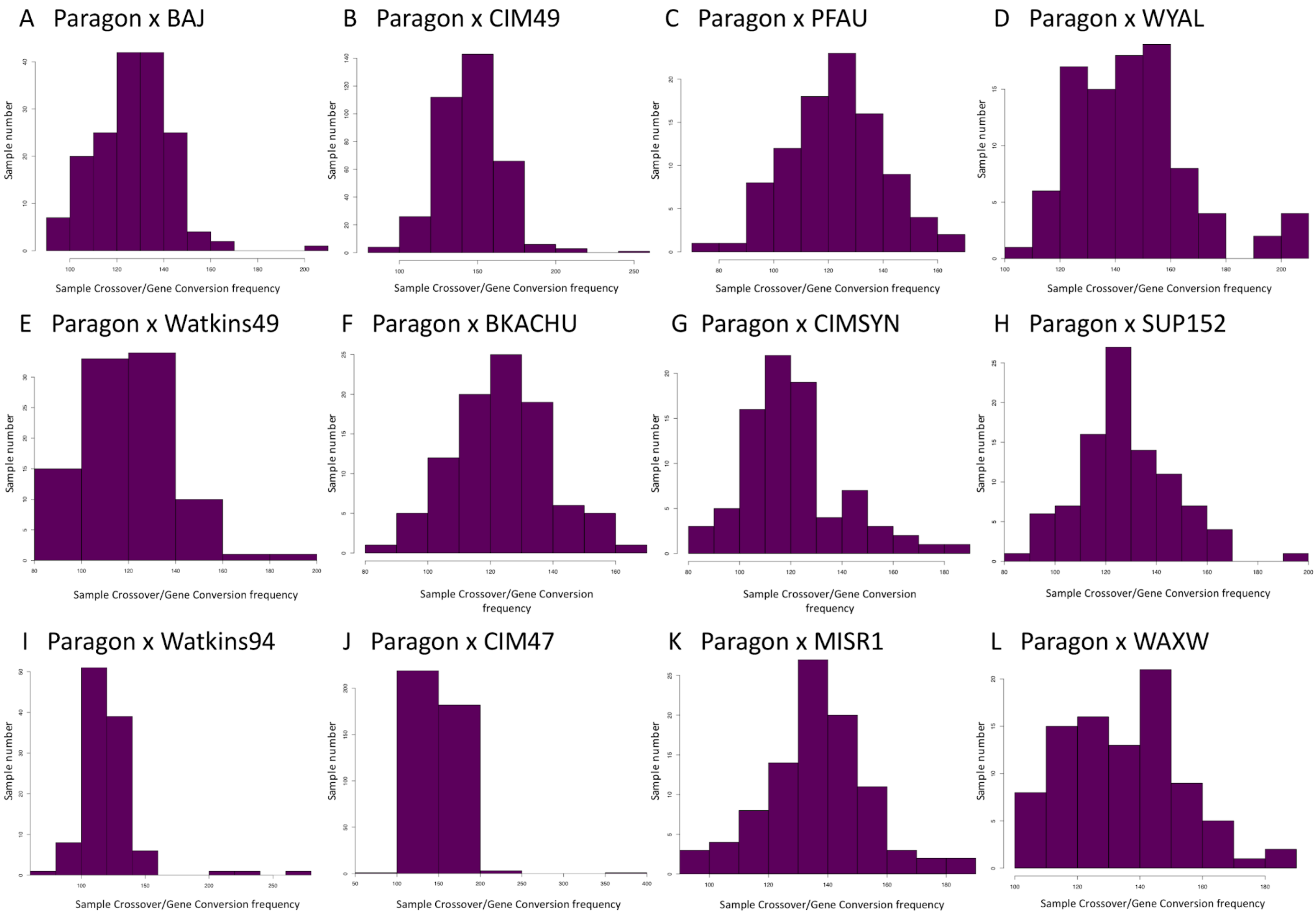

Figure S5. Frequency of COs and GCs per RIL. For each of the populations under analysis figures A-L represent the number of COs and GCs i.e. total sequence exchange events recorded for each RIL (CO frequency per sample) as a frequency histogram.

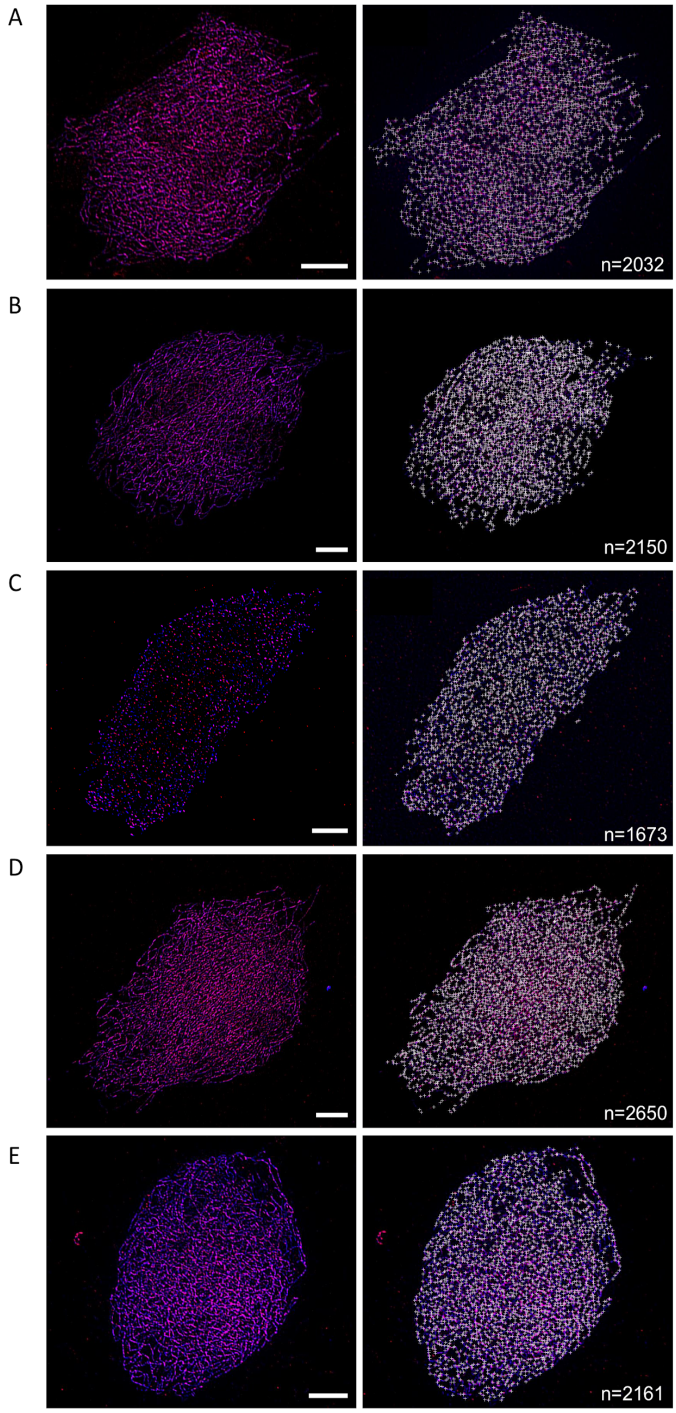

Figure S6. Defining DSBs in hexaploid wheat. Immunolocalisation of the chromosome axis protein ASY1 (blue) and γH2A.X (red) a marker for DNA double strand breaks on hexaploid wheat leptotene male meiotic nuclei. (A-E) five individual replicate nuclei with the image to the left the original nuclei and the rightmost image the original nuclei with γH2A.X foci marked that co-localise with ASY1. Scale bar = 10µM. Mean γH2A.X foci = 2133 (n=5), SD = 313, SE = 157.

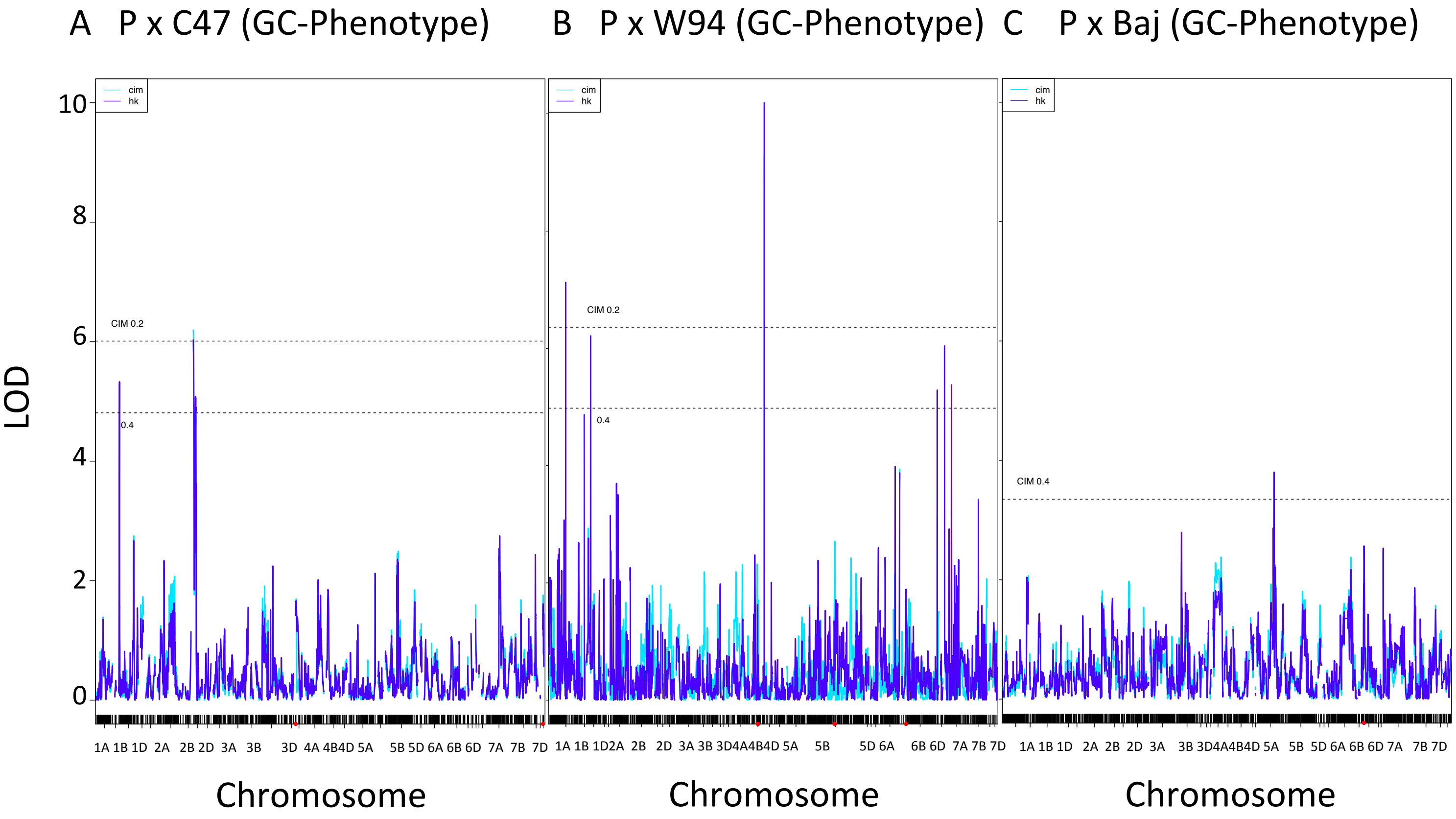

Figure S7. Output from additional QTL analysis. QTL analysis output for the additional three populations that yielded significant associations for GC-Phenotype ($p<0.05$). Detailing LOD scores plotted over the respective linkage groups i.e. chromosomes.

A

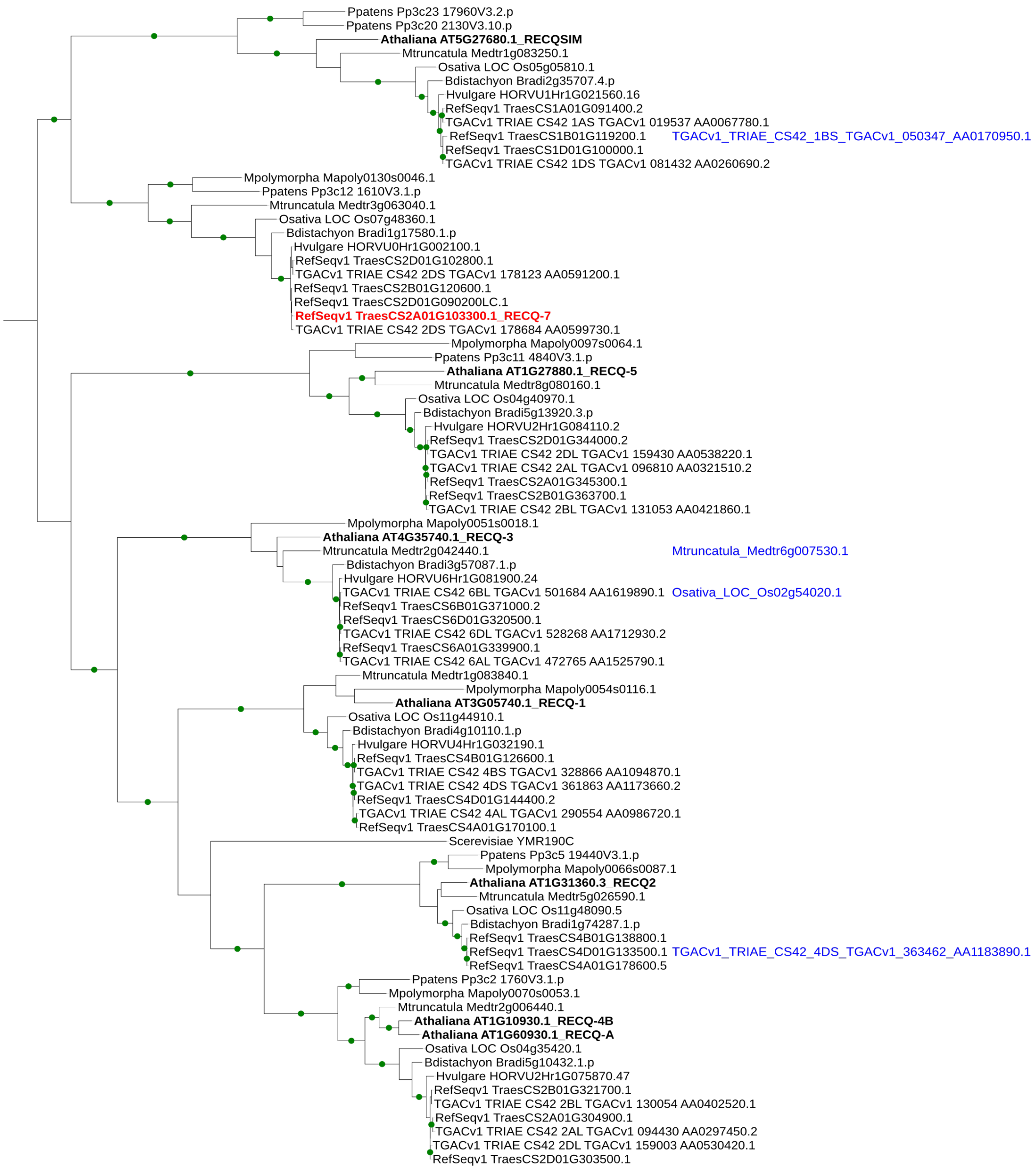

Figure S8. Phylogenetic Analysis of the *RecQ* Gene family. (A) Phylogenetic tree of all *RecQ*-like genes across ten species with sequence similarity to the RecQ helicase family (Methods). Bootstrap values >= 90 % are shown as green dots on the branches. Additional gene identifiers shown in blue are truncated genes belonging to the RecQ family at the position indicated, based on a BLASTP search of the Helicase_C family.

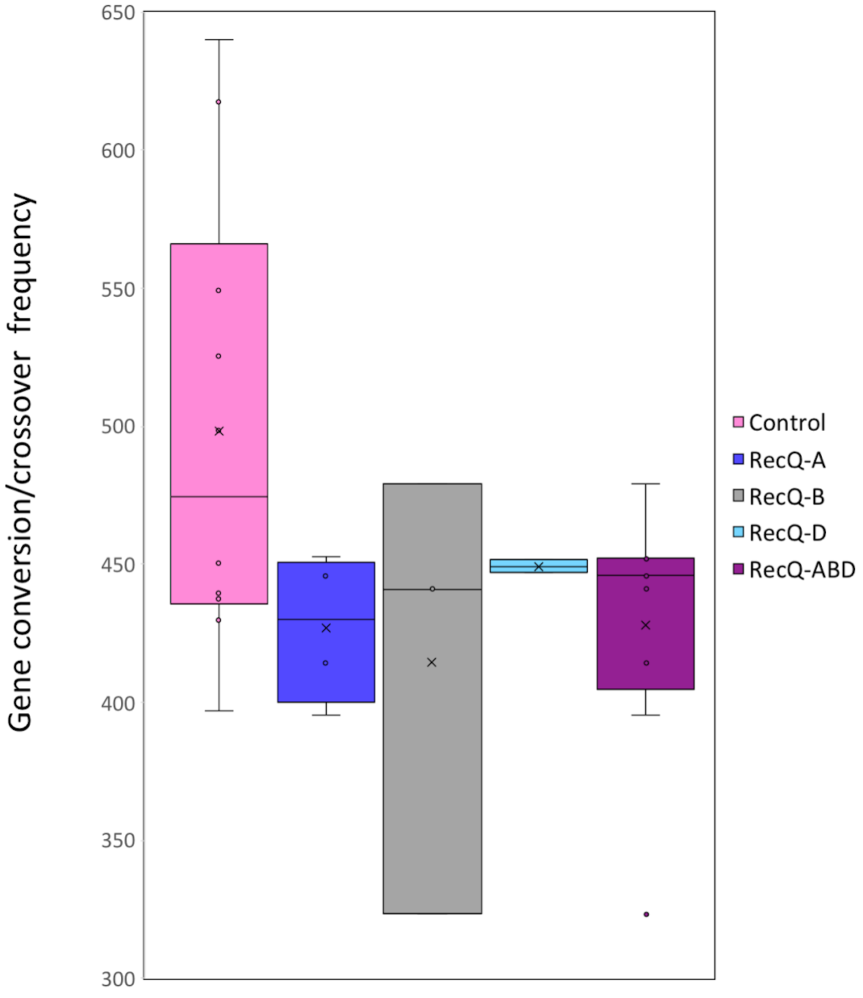

Figure S9. Examination of homoeologs of candidate genes from QTL analysis. Box plot comparison of the knockout *RecQ-7* lines with the control lines, defining CO/GC frequency using GC-Phenotype. Knockouts are shown for the candidate *RecQ-7* gene on chromosome 2A (RecQ-A) and its predicted homoeologs on chromosome 2B (RecQ-B) and 2D (RecQ-D). The RecQ-D group also includes knockouts for the low confidence gene on chromosome 2D that also shows high similarity to the homoelogous gene trio. Finally, all knockouts, independently of which homoeologous gene is knocked out, are pooled into a single group for comparison (RecQ-ABD).

Supplemental Tables

Table S1. Detailing the parental founders of the 13 populations that were used in this study.

Parent 1 name	Parent 1 information	Parent 2 name	Parent 2 information
Paragon	UK elite line	Chinese Spring	Landrace (reference variety)
Paragon	UK elite line	Watkins 1190049	Watkins Bread wheat landrace diversity collection
Paragon	UK elite line	Watkins 1190094	Watkins Bread wheat landrace diversity collection
Paragon	UK elite line	BAJ	CIMMYT Breeders line
Paragon	UK elite line	BKACHU	CIMMYT Breeders line
Paragon	UK elite line	CIMMYT 47	CIMMYT Breeders line
Paragon	UK elite line	CIMMYT 49	CIMMYT Breeders line
Paragon	UK elite line	CIMMYT SYN	CIMMYT Breeders line
Paragon	UK elite line	MISR1	CIMMYT Breeders line
Paragon	UK elite line	PFAU	CIMMYT Breeders line
Paragon	UK elite line	SUP152	CIMMYT Breeders line
Paragon	UK elite line	WAXW	CIMMYT Breeders line
Paragon	UK elite line	WYAL	CIMMYT Breeders line

Table S2. Population statistics for the 13 populations under investigation. Detailing for each of the 13 populations; population size, the number of 35K array SNPs that were consistently assayed across the population and informative i.e. polymorphic between Parent 1 and Parent 2, the number of unique COs identified per sample, the average number of COs per sample and the average number of GCs per sample.

**Parent 1 name**	**Parent 2 name**	**Population Size**	**Number of 35K SNPs assayed**	**Unique COs in population**	**COs per sample**	**GCs per sample**
Paragon	Chinese Spring	269	8369	466	51.9	335.5
Paragon	Watkins 1190049	94	4010	357	43.5	75.9
Paragon	Watkins 1190094	108	4057	383	46.7	73.4
Paragon	BAJ	168	3765	354	47.8	79.1
Paragon	BKACHU	94	3884	343	42.7	80.4
Paragon	CIMMYT 47	406	4789	394	50.9	97.9
Paragon	CIMMYT 49	361	4502	407	43.8	101.7
Paragon	CIMMYT SYN	83	4021	332	40.8	79.9
Paragon	MISR1	94	4391	340	45.6	89.2
Paragon	PFAU	94	4629	342	45.5	76.7
Paragon	SUP152	94	4075	345	45.9	81.1
Paragon	WAXW	90	4085	329	45.1	88.4
Paragon	WYAL	94	4618	389	48.1	96.6

Table S3. Shared COs within the 13 populations under investigation. Detailing for each of the 13 populations; the percentage of the total number of COs that are shared between various numbers of RILs.

Sample Number	% COs shared (466 total) PxCS	% COs shared (357 total) PxWat49	% COs shared (383 total) PxWat94	% COs shared (354 total) PxBAJ	% COs shared (343 total) PxBKACHU	% COs shared (394 total) PxCIM47	% COs shared (407 total) PxCIM49	% COs shared (332 total) PxCIMSYN	% COs shared (340 total) PxMISR1	% COs shared (342 total) PxPFAU	% COs shared (345 total) PxSUP152	% COs shared (329 total) PxWAXW	% COs shared after bp collapse (389 total) PxWYAL
1	6.65	15.4	10.4	7.1	14.6	3.8	6.1	12.3	12.4	11.7	9.0	12.2	10.8
2	6.44	10.9	8.4	5.4	8.7	4.8	5.2	11.7	9.7	7.0	6.4	4.9	10.0
3	7.51	8.1	7.0	5.4	8.7	2.5	3.4	8.4	7.6	7.9	8.7	7.3	9.0
4	5.58	7.0	9.7	4.0	6.1	3.6	3.7	6.6	6.5	6.1	5.2	7.6	9.8
>=2	93.3	84.6	89.6	92.9	85.4	96.2	93.9	87.7	87.6	88.0	91.0	87.8	89.2
>=5	73.8	58.5	64.5	78.2	61.8	85.3	81.6	60.8	63.8	67.0	70.7	68.1	60.4
>=10	58.6	40.6	46.7	61.3	43.1	70.6	62.9	41.9	48.2	48.8	48.4	48.3	41.1
>=20	43.6	22.1	24.0	43.5	23.3	57.4	46.9	16.3	25.3	24.0	24.6	23.4	22.6
>=30	34.1	12.9	13.1	31.6	9.9	46.4	36.6	5.7	9.4	10.8	9.9	10.9	12.1
>=40	29.0	2.5	7.0	21.5	2.9	43.1	33.7	0	2.9	2.0	1.7	3.3	1.3
>=50	24.5	0.3	1.8	13.6	0.6	39.6	30.7	0	0.3	0	0	0	0
>=100	6.0	0	0	0	0	19.3	13.0	0	0	0	0	0	0
>=125	2.6	0	0	0	0	13.5	6.6	0	0	0	0	0	0
>=150	0	0	0	0	0	9.6	3.7	0	0	0	0	0	0
>=200	0	0	0	0	0	0.3	0	0	0	0	0	0	0

Table S4. Shared COs between the 13 populations under investigation. Detailing the percentage of the total number of COs that are shared between 1-13 populations according to how many RILs within specific populations have the CO. Cumulative percentage totals for each row equal 100%.

In >=1 popln the min no of samples brkpt is in	% windows with brkpts in 1 popln	% windows with brkpts in 2 poplns	% windows with brkpts in 3 poplns	% windows with brkpts in 4 poplns	% windows with brkpts in 5 poplns	% windows with brkpts in 6 poplns	% windows with brkpts in 7 poplns	% windows with brkpts in 8 poplns	% windows with brkpts in 9 poplns	% windows with brkpts in 10 poplns	% windows with brkpts in 11 poplns	% windows with brkpts in 12 poplns	% windows with brkpts in 13 poplns
1	9.5394	7.7302	6.5788	4.7697	4.7697	6.25	5.09868	3.4539	7.0723	7.40131	7.23684	10.8552	19.243
2	7.7186	7.0325	6.6895	4.2881	4.8027	6.51801	5.31732	3.6020	7.3756	7.71869	7.54716	11.3207	20.068
5	6.1302	4.9808	4.9808	4.0229	4.0229	6.5134	5.55555	3.6398	8.2375	8.62068	8.23754	12.6436	22.413
10	5.7649	4.43	4.656	3.3259	3.3259	5.76496	4.8780	3.547	7.5388	9.09090	7.76053	13.9689	25.942
25	5.2478	3.4985	4.0816	2.6239	2.9154	4.95626	3.79008	3.7900	8.1632	8.74635	6.70553	13.994	31.487
50	5.0847	0.8474	2.5423	0.8474	0.8474	5.08474	3.38983	4.6610	10.169	9.74576	5.9322	13.9830	36.864

Table S5. Samples selected for whole genome skim sequencing. Detailing the samples sequenced from the Paragon x Chinese Spring population, the number of COs defined for each sample and the number of GCs defined for each sample (those classed as high are in bold type and low are underlined; see Methods for details). Also detailing the number of sequencing reads aligned to the IWGSC RefSeqV1 Chinese Spring reference genome plus average depth and percentage of genome mapped. Finally, the number of SNPs called are detailed alongside the number of Paragon-Chinese Spring parent specific sites that have sequence information in the skim sequenced samples.

RIL Name (Paragon x Chinese Spring population)	CO frequency (35K array)	GC frequency (35K array)	Aligned sequencing reads-post filtering	Average depth of coverage	% of reference genome mapped	Number of SNPs identified	Number of Paragon-CS specific sites with information (31,327,143 total)
0055	**73**	**429**	617,259,125	6.74	92.21	13,984,026	29,182,483
0334	**70**	**507**	577,017,395	6.33	91.21	19,739,698	27,012,789
0200	54	**461**	632,964,899	6.91	91.76	19,285,179	28,830,075
0270	54	**483**	623,216,599	6.73	92.05	14,937,252	28,278,916
0008	**65**	413	553,203,920	6.16	90.65	26,542,822	27,609,483
0119	**69**	409	602,689,927	6.65	91.43	20,542,948	27,779,818
0005	55	363	581,127,334	6.39	90.91	23,687,781	27,926,026
0018	52	386	496,591,667	5.48	90.91	17,775,994	27,712,617
0097	53	<u>322</u>	651,920,209	7.20	90.69	29,376,821	28,166,225
0004	<u>34</u>	378	483,449,595	5.37	90.40	18,188,963	27,408,941
0010	<u>37</u>	<u>319</u>	559,978,269	6.22	90.39	21,867,247	27,831,695
0281	<u>39</u>	<u>306</u>	629,503,914	6.82	92.36	14,797,624	29,149,453

Table S6. Validation of CO frequency QTLs from Paragon x Chinese Spring population using the Cadenza TILLING population. Detailing; candidate gene, length of CDS, TILLING line with likely effector mutation in candidate gene, consequence of mutation (stop codon or missense mutation), from exome sequencing of line the coverage of the reference with wild type reads (WT) and mutant allele containing reads (Mut).

Candidate gene	Length of CDS (bp)	Annotation	TILLING Line	Category	Mutation Consequence	WT Coverage	Mut Coverage	CDS Position
TraesCS2A01G103300	2679	Chromosome 2A ATP-dependent helicase RecQ (*RecQ-7)*	Cadenza0927	het5hom3	stop_gained	28	11	228
			Cadenza1716	het5hom3	stop_gained	0	18	940
			Cadenza0560	het5hom3	stop_gained	14	21	1318
			Cadenza1279	het5hom3	stop_gained	6	8	1758
TraesCS2B01G120600	2607	Chromosome 2B *RecQ-7* B homoeolog	Cadenza1733	het5hom3	stop_gained	17	12	130
			Cadenza0393	het5hom3	stop_gained	19	11	1307
			Cadenza1675	het5hom3	stop_gained	0	33	1887
TraesCS2D01G102800	2682	Chromosome 2D *RecQ-7* D homoeolog	Cadenza0207	-	stop_gained	0	8	205
TraesCS2D01G090200	306	Chromosome 2D *RecQ-7* D homoeolog	Cadenza0284	-	stop_gained	13	13	172
TraesCS6A01G026300	2112	Chromosome 6A Holliday junction ATP-dependent DNA helicase RuvB	Cadenza1616	het5hom3	stop_gained	0	2	289
			Cadenza1495	het5hom3	stop_gained	17	18	787
			Cadenza1530	het5hom3	Splice_acceptor_variant	0	4	
			Cadenza2095	het5hom3	missense_variant	34	4	1162
			Cadenza0129	het5hom3	missense_variant	7	6	82
			Cadenza1709	het5hom3	missense_variant	0	35	443
			Cadenza0962	het5hom3	missense_variant	0	9	458
			Cadenza1410	het5hom3	missense_variant	67	51	476
TraesCS2A01G304900	3423	Chromosome 2A *Arabidopsis RecQ4A/4B* homolog	Cadenza1636	het5hom3	stop_gained	0	3	1577
			Cadenza0387	het5hom3	stop_gained	11	13	1624
			Cadenza1371	het5hom3	stop_gained	53	48	3325
			Cadenza0861	het5hom3	stop_gained	14	10	3363
			Cadenza0300	het5hom3	stop_gained	25	8	257

			Cadenza0213	het5hom3	stop_gained	0	11	258
			Cadenza0297	het5hom3	stop_gained	25	12	1513
			Cadenza0865	het5hom3	stop_gained	0	51	1585
TraesCS2B01G321700	3579	Chromosome 2B *Arabidopsis RecQ4A/4B* homolog	Cadenza1575	het5hom3	stop_gained	0	12	463
			Cadenza0586	-	stop_gained	0	57	1575
			Cadenza1993	-	stop_gained	83	70	1774
			Cadenza0250	-	stop_gained	0	29	3286
TraesCS2D01G303500	3606	Chromosome 2D *Arabidopsis RecQ4A/4B* homolog	Cadenza0668	het5hom3	stop_gained	0	23	148
			Cadenza0947	het3hom2	stop_gained	1	6	258
			Cadenza1267	het5hom3	stop_gained	8	5	1213
			Cadenza1178	-	stop_gained	0	26	1761
			Cadenza1700	-	stop_gained	23	5	2574
			Cadenza0316	-	stop_gained	15	17	3546

Table S7. Validation of CO frequency QTLs from various populations using the Cadenza TILLING population. Detailing; candidate gene, length of CDS, TILLING line with likely effector mutation in candidate gene, consequence of mutation (stop codon or missense mutation), from exome sequencing of line the coverage of the reference with wild type reads (WT) and mutant allele containing reads (Mut).

Candidate gene	Length of CDS (bp)	Annotation	TILLING Line	Category	Mutation Consequence	WT Coverage	Mut Coverage	CDS Position
TraesCS2B01G103300	-	Chromosome 2B PIF2	Cadenza1346	het5hom3	missense_variant	8	10	1049
			Cadenza0750	het2hom2	missense_variant	1	3	914
TraesCS5A01G257000	1635	Chromosome 5A WPP	Cadenza0871	het5hom3	missense_variant	9	15	763
			Cadenza0104	het5hom3	missense_variant	0	23	142
			Cadenza0190	het5hom3	missense_variant	14	10	625
			Cadenza0453	het5hom3	missense_variant	14	15	616
			Cadenza0706	het5hom3	missense_variant	4	6	592
			Cadenza2048	het5hom3	missense_variant	4	5	532
			Cadenza2010	het5hom3	missense_variant	20	4	497
TraesCS4B01G006700	2994	Chromosome 4B HIRA	Cadenza1457	-	stop_gained	0	39	175
			Cadenza0142	-	stop_gained	16	16	553
			Cadenza0627	-	stop_gained	3	6	553
			Cadenza1024	-	stop_gained	0	21	582
			Cadenza1720	-	stop_gained	4	5	910
			Cadenza0406	-	stop_gained	0	19	2035
